# 

**Published:** 1891-07

**Authors:** 


### GENERAL INDEX VOL. VII.

Contents July, 1891, to June, 1892, Inclusive.

Ætiology and Treatment of Tetanus.................... 1
Austin District Medical Society, 223, 250, 363, 189, 100, 14, 100
Amputation of the Cervix Uteri........................ 14
Alkaloids, a plea for the more general use of............. 39
Albuminuria, practical points on management........... 45
Antipyretics, a country doctor's experience with.......... 52
Association American Medical Editors.................. 56
American Medical Editors, Association of............... 56
A New and Remarkable Phase in Medical Practice....... 59
After-pains, How to Prevent.......................... 213
A Feast of Reason, Austin District Medical Society....... 223
Account of Killing of Dr. Reeves..................... 257
Absurd and Dangerous Prescriptions.................. 292
Antikamnia (opposed to pain)......................... 344
Abortions, Management.............................. 351
A Higher Professional Standard....................... 365
Amick, Dr. and the *Lancet-Clinic*..................... 370
A Medical Secretary of Public Health..............142, 371
An Unpardonable Liberty............................ 373
Aided Intestinal Suture, Some Observations on the Expermental Use of.............................. 157
Alcohol Not a Stimulant............................ 187
A Bitter Pill....................................
American Medical Association, Membership in........... 100
A Modern Waterloo................................ 142
A Discrepancy Explained............................ 109
A "Canser" Doctor................................ 110
Aurora Epidemic, The.............................. 116
Aurora Epidemic, Discussion on....................... 127
As Clear as Mud.................................. 146
A New Feature in Medical Teaching.................... 147
Amputation (double) of Lower Extremity................ 355
Anachronism, The Prescription Druggist on ........... 289
A National Family Paper............................ 194
Appointment of Lectures in Texas Medical College ...... 194
Abdomen, Pus in, Ten cases.......................... 77
American Medical Association, Membership in........... 100
A Private Hospital for Austin........................ 266
A Disgraceful Swindle—"The Ladies' Friend"........ .. 270
Address by Dr. Paine at Commencement University of Texas, Medical Department........................ 404
Acne, ℞ for...................................... 451

Barnes' Caoutchouc Dilator, some Modifications of ....... 309
Book Notices and Reviews ..............347, 381, 304, 69, 417
Banquet, The A. D. M. S ........................ 225
Biographical Sketch of Dr. J. D. Osborn ............... 408
Biographical Sketch of Dr. Reeves ..................... 263
Biographical Sketch of Dr. F. S. White................ 265
Bladder, Stab Wound in........................ ...... 88
Burke, Dr. T- S., Death of............................ 150
Crocodile Tears.................................. 415
Commencement Exercises Texas Medical College ........ 403
Circumscribed Dropsy ................................ 137
Chloralamid in Surgery............................. 12
Cherokee County Medical Society ...................... 24
Contract Surgeons in Relations to Railroads............. 29
Cancer, its Causes and Treatment...............215, 284, 173
Capiat, The....................................175, 190
Cost of Transactions, Comparative..................... 399
Conservative Laparotomy ............................... 386
Corns and Other Things .............................. 409
Country Doctors' (A) Experience with Antipyretics ...... 52
Chronic Supurative Otitis ............................ 199
Can Man have Milk-Leg? ............................ 248
Cerebral Hemorrhage; Trephining for .................. 275
Central Texas Medical Association—Program........... 287
Clinical Notes from N. Y. Hospital.................... 325
Correspondence.. ...... ............325, 217, 208, 284, 181
"Cure" for Consumption; The Latest.................. 333
Cause of Cattle Fever--Ticks the .... ............... . 335
Cervix Uteri, Amputation of, Discussion on ............. 14
Comparative Cost of Reprints........................ 110
Chloroform, death from ...... ....................... 442
Castration for Criminals. . ............ ........... 448
Christian Science (?)........... ............... .... 440
Code of Ethics, the ............ ....... ............ 437
Cystotomy, Supra-pubic.......................... 439
Diabetes; Its Causes and Treatment ......... .......... 196
Discrepancy (A) Explained.......................... 109
Doctors (The) Reciprocity....................... .... 300
Death of Dr. D. Hays Agnew ........ .... ........... 378
Dr. Paines Address.................................. 404
Dr. Clopton's Address ............................... 402
Dr. J. D. Osborn's Address........... ........... ... 408
Double Amputation of Lower Extremity.......... ... 257
Dr. Amick and Lancet Clinic ........................ 370
Discussion on Hysteria............................. 168
" " New Operation for Hernia ................ 14
" " Empyema.................................. 14
" " Pessaries ................................. 97
" " Aurora Epidemic ......................... 127
" " Syphilis .................................. 241
" " Fevers.................................... 250

"   " Amputation on Cervix Uteri .............. 14
"   of Dr. Keiler's Paper.... ...................... 314
"   " Dr. Parker's Paper....... ...................... 254
Distention of Gall Bladder—Operation.................. 278
Dietetic Treatment of Disease.......................... 222
Domestic Animals vs. Man.............................. 226
Danforth, Dr. Grace; Letter from........................ 248
Dr. West' Criticism, Reply to .......................... 130
Deaths from Chloroform ................................ 442
Ehrlenmeyer's Method................................ 367
Enteritis Simulating Obstruction of Bowels ............. 204
Empyema, Discussion on............................... 14
Exploits in Medicine ................................ 139
Election of Professors Texas Medical Collage ........... 67
Essentials of Anatomy ................................ 69
Epistaxis, an Easy Method of Plugging in............... 399
Fœtal Skeleton Passed per Rectum....................... 217
Faculty (The) of Medical Department University of Texas 101
Greeting, New Year.................................... 228
Give it a Name ...................................... 264
How we Treat Wounds To-day ........ ............. 9
Homœopathy with a Whisky Cure Annex .............. 369
Hysteria ............................................ 163
How to Prevent After-pains ............................ 213
Hernia, New Operation for .............................. 14
How I have Dealt with Ten Cases Pus in Abdomen ...... 77
Higher Education..................................... 67
Heredity Health and Beauty ............................ 69
Homopathic Demonstration; a Rousing?.................. 410
Is Syphilis Transmitted from Father to Child............ 233
Influenza; Ætiology, Symptoms and Treatment..........280-1
Intestinal Obstruction (acute) Simulating Enteritis....... 204
International Clinics................................. 95
International Medical Journal and Practitioners Index.... 196
Insane, What to do with the..... ...................... 85
Journalistic Lagniappe................................ 103
Lucillia Marcillaria or Texas Screw Worm ............. 247
Laparotomy for stab wound in bladder.................. 88
La Grippe, ℞ for .... .............................. 299
Lancet-Clinic and the Amick Nostrum ................. 370
Lecturers appointed in Texas Med. College ............ 194
Libel Suit in England ................................ 446
Mississippi Medical Monthly........................... 190
More Quackery ....................................... 183
Morris, Dr. Seth M .................................. 413
Medical Department University of Texas, commencement. 403
Medical and Surgical Gynecology, treatise on ........... 417
Membership in American Medical Association ........... 100
Married, Dr. Bibb .................................... 229
Married, Dr. Kingsly .................................. 411

Medical Department University of Texas ............... 402
Medical Societies, new county ........................ 56
Mississippi Valley Medical Association ................ 58
Medical News and Miscellany ......................... 267
Microscope, the value of the, in diagnosis ............. 243
Microscope, the, in Medicine ......................... 169
Morphine Habit, treatment of ......................... 321
Modifications (some) of Barnes' Uterine Dilators ........ 309
McClelland's Reginal Anatomy, review of .............381-304
Management of Abortions, the ........................ 251
Medical News and Miscellany ...................... 448-452
North Texas Hospital for the Insane, report of 301 ...... 301
Notes of the Tyler Meeting .......................... 396
Novel Treatment of Pthisis proposed .................. 182
Northwest Texas Medical Society ...................... 184
National Board of Health ............................ 227
New York Post Graduate Hospital Report ............... 302
New Year Greeting ................................. 228
National Board of Health, bill for a ................... 227
Osborn, Dr. J. D. (portrait), biography of .............. 408
Ophthalmic discs .................................. 107
Private Hospital, a, for Austin ....................... 266
Publisher's Notes......72, 153, 272, 306, 243, 384, 232, 38, 420
197, 112.
Prescription Druggist, the, an Anachronism ............. 289
Pan American Medical Congress............285 286, 361, 181
Perityphlitis ...................................... 315
Principles and Practice of Medicine, treatise upon ....... 419
Polypi of Middle Ear cause of C. Sup. Otitis ........... 199
Plains Medical Society .............................. 57
Pure Phosphorus in Diseases of Nervous System ......... 48
Poeta Nascitur Non Fit .............................. 192
Pus in Abdomen, ten cases ........................... 77
Pott's Disease, Wiring of Vertebra in .................. 90
Pessaries, Discussion on, ..........................96, 97
Preserving Bodies for Dissection ...................... 425
Purulent Pleurisy .................................. 434
Peritonitis, Tubercular .............................. 430
Rousing (a) Homeopathic Demonstration ................ 410
Retrench or Bankrupt ............................... 399
Removal of the Secundines, the Capiat ................. 175
Reginal Anatomy, McClelland's .....................381, 304
Rockwall County Medical Society ...................... 285
"Regulating the Practice" ........................... 144
Reeves, Dr.; the Killing of .......................... 263
Reeves, D.; Biography of ............................ 263
Report of North Texas Hospital for the Insane .......... 301
Report of N. Y. Post Graduate Hospital and Babies Ward 302
Richardson, Dr. T. G., death of ....................... 451
Sim, Dr. F. L ..................................... 298

Sad Case, A; Death of Dr. Cook ........ 232
Thompson, Prof. J. E ........ 270
The "Ladi's Friends" ........ 270
The Prescription Druggist—An Anachronism ........ 289
Stories of a Country Doctor ........ 107
Syphis (Is) Transmitted to Child ........ 233
Syphillis; Discussion on ........ 241
Southeast Texas Medical Society Revived ........ 364
Screw Worms in the Nose.—Dr. Osborn ........ 220
Screw Worm in the Nose.—Dr. T. J. Turpin ........ 221
Smith County Medical Society ........ 221
Supra-Pubic Lithotomy—A Case ........ 54
Some Practical Observations on Management of Albuminuria in Pregnancy ........ 45
Sat Down Upon ........ 407
Synopsis of Proceedings of the T. S. M. Association ... 389
Some Observation on Aided Intestinal Suture ........ 157
Secundines; Removal of, Capiat ........ 175
Sexual Neurosthenia ........ 195
Strain, Dr. T. W.; Death of ........ 192
Southern California; Climate of ........ 192
Stab-wound in Bladder ........ 88
Selected Formulæ ........ 443
Supra-Pubic Cystotomy ........ 439
Tubercular Peritonitis ........ 430
The Substitutor ........ 445
The New Texas Medical College ........ 27
The Relation of Contract Surgeons of Railway Corporations to the General Profession ........ 29
The Tick Theory ........ 372
The Bug under the Chip ........—... 30
Typhlitis; Discussion on ........ 14
Tyler Meeting; The ........ 389
" " Notes of ........ 396
The Youth's Companion ........ 194
Treatment Wanted ........ 193
Texas Sanitarian; The ........ 108
Tetanus; Rational Treatment of ........ 1
Ticks and Texas Fever ........ 372, 357
Treatment of Morphine Habit ........ 321
Texas State Medical Association ........ 329, 25
The New Superintendent State Lunatic Asylum .... 265
The Asylum Tragedy ........ 257
Texas Screw Worm ........ 247
Treatment of Trachoma by Expression ........ 283
Trephining for Cerebral Hemorrhage ........ 275
The Latest "Cure" for Consumption ........ 333
Ticks, as Cause of Cattle Fever ........ 335
Useful Formulæ ........ 424
Vertebra, Wiring of the,—Hadra ........ 93

What to Do with the Insane . . . . . . . . . . . . . . . 85
Wiring of the Vertebra as a means of Immobilization . . 90
Waterloo, a Modern . . . . . . . . . . . . . . . . . . . 142
When Lovely Woman Stoops to Folly . . . . . . . . . 152
White, Dr. F. S., The New Superintendent . . . . . . 265
Watching the Watchman . . . . . . . . . . . . . . . . 338
Wooing (A) to the Woods . . . . . . . . . . . . . . . 416

---

### Contributors to Vol. 7, July, 1891, to June, 1892.

Beall, Dr E J, Ft Worth.
Bennett, Dr T J, Austin.
Blailock, Dr W R. McGregor.
Baldinger, Dr W H, Galveston.
Cummings, Dr J, Austin.
Cunningham, Dr J L, Ft Worth.
Church, Dr B F, Terrell.
Crofford, Dr T J, Memphis, Tenn
Carhart, Dr J W, Lampasas.
Clark, Dr Eugene, Lockhart.
Davis, Dr W B, Pueblo, Colorado.
Danforth, Dr Grace, Chicago, Ill.
Eaves, Dr J F, Millican.
Fox, Dr Sam J, Willis.
Gregg, Dr R S, Manor.
Hill, Dr H B, Austin.
Hadra, Dr B F, Galveston.
Hulen, Dr Vard H, Galveston.
Hodges, Dr R C, Galveston.
Johnson, Dr James, Brownwood.
Keiller, Prof Wm F, R S C Ed, Galveston.
Lee, Dr Geo H, Galveston.
Lanphear, Prof E, Kansas City, Mo.
Leake, Dr Fannie, Austin.
Menger, Dr R, San Antonio.
McGee, Dr D B, Brownwood.
Nowiersky, Dr B J, Cestohowa.
Powell, Dr W P, Willis.
Paine, Prof J F Y, Galveston.
Parker, Dr Daniel, Calvert.
Price, Dr J Sam'l, Beaumont.
Poyner, Dr J S, Bartlett.
Rogers, Dr B A, Georgetown.
Reeves, Dr C S, Lone Grove.
Slawianski, Prof, St Petersburg, Russia.
Turpin, Dr T J, Laredo.
White, Dr F S, Austin.
Wooten, Dr T D, Austin.
Weller, Dr C O, Austin.

